# Handgrip Strength: Associations with Clinical Variables, Body Composition, and Bone Mineral Density in Adults with Cystic Fibrosis

**DOI:** 10.3390/nu13114107

**Published:** 2021-11-16

**Authors:** Victoria Contreras-Bolívar, Casilda Olveira, Ignacio Ruiz-García, Nuria Porras, Maria García-Olivares, Francisco José Sánchez-Torralvo, Maria Victoria Girón, Silvia P. Alonso-Gallardo, Gabriel Olveira

**Affiliations:** 1 Servicio de Endocrinología y Nutrición, Hospital Regional Universitario de Málaga, Instituto de Investigación Biomédica de Málaga (IBIMA), Universidad de Málaga, 29010 Málaga, Spain; victoriaconbol@gmail.com (V.C.-B.); nach1991@gmail.com (I.R.-G.); nporrasperez@hotmail.com (N.P.); mery.garcia.96@gmail.com (M.G.-O.); fjstsol@gmail.com (F.J.S.-T.); silvia_94_86@hotmail.com (S.P.A.-G.); 2 Servicio de Endocrinología y Nutrición, Hospital Universitario Clínico San Cecilio, Instituto de Investigación Biosanitaria (Ibs, Granada), 18016 Granada, Spain; 3 Servicio de Neumología, Hospital Regional Universitario de Málaga, Instituto de Investigación Biomédica de Málaga (IBIMA), Universidad de Málaga, 29010 Málaga, Spain; casi1547@separ.es (C.O.); marivi_giron@hotmail.com (M.V.G.); 4 Centro de Investigación Biomédica en Red-Diabetes y Enfermedades Metabólicas Asociadas (CIBERDEM), 08036 Barcelona, Spain

**Keywords:** cystic fibrosis, handgrip strength, Jamar handgrip dynamometry, malnutrition, nutritional assessment

## Abstract

Background: Loss of fat-free mass (FFM) is associated with an increase in morbidity and mortality in cystic fibrosis (CF) patients. Handgrip strength (HGS) measures muscle function and may be associated with clinical parameters with prognostic value. Our objectives were to evaluate muscle strength through HGS in CF patients and to determine if there are any associations with respiratory clinical variables, FFM, and bone mineral density (BMD). Methods: A cross-sectional study conducted in clinically stable patients. We evaluated muscle function through HGS, respiratory function—forced expiratory volume in 1 s (FEV1) (%), forced vital capacity (FVC) (%), bronchorrhea, annual exacerbations, and body composition (FFM and FFM index, FFMI: fat-free mass in kg/height in m^2^) and Bone Mineral Density (BMD) through densitometry (DXA). Results: The study included 53 CF patients (58.5% females, mean age 28.3 ± 8.1, body mass index (BMI) 21.7 ± 3.4). The mean values for dynamometry were 40.2 ± 8.1 kg in males and 23.1 ± 7.0 kg in women, being 20.8% below the 10th percentile. Patients with lower muscle strength showed significantly more exacerbations and lower FEV1% and FVC%, as well as lower BMI, worse BMD (g/cm^2^), T-score, and Z-score. A significant and positive correlation was found between the mean and maximum dynamometry values and age, FVC%, BMI, FFMI, FFM (kg), and BMD. Conclusions: For adults with CF, HGS is a practical tool for assessment of health status. Low values reflect poor nutritional status and are associated with poor respiratory function, low fat-free mass and low bone mineral density.

## 1. Introduction

Cystic fibrosis (CF) is a multisystem disease caused by the alteration of a unique gene located on chromosome 7 (CFTR, cystic fibrosis transmembrane conductance regulator gene). Malnutrition behaves as a risk factor predictor of morbidity and mortality [1]. Until a few years ago, CF was considered to be associated with malnutrition because it was practically always present at the time of diagnosis and the vast majority of patients suffered a deterioration of their nutritional status and died very malnourished. Currently, the prevalence of malnutrition has decreased significantly, although figures close to 25% continue to be reported in both children and adults [2]. Malnutrition is associated with a decrease in muscle mass, which translates to impaired functional tests and worse body composition. The multisystem involvement in CF, with progressive clinical impairment, may result in loss of peripheral skeletal muscle mass, a progressive decrease in muscle strength, physical activity limitations, and a reduction in activities of daily living, with deterioration in quality of life [3,4].

Clinical practice CF guidelines recommend performing a periodic nutritional assessment [1]. At every visit, patient weight, height, body mass index (BMI), and weight loss over time are recorded. Besides BMI, it is also recommended to evaluate body composition through different techniques in order to estimate fat-free mass (FFM) [5]. Handgrip strength (HGS), a measure of muscle function, is used as a surrogate for muscle mass and is an indicator of nutritional status. A low level of muscle strength worsens health status and malnutrition [6] and is strongly associated with other clinically and prognostically important measures [7]. Recently, the Global Leadership Initiative on Malnutrition (GLIM) criteria for diagnosing malnutrition have been published, including within the phenotypical criteria muscle mass reduction through such validated techniques as DXA (Dual-energy x-ray absorptiometry), bioelectrical impedance analysis, computed tomography, or magnetic resonance imaging. However, some of these methods for measuring body composition are often unreliable, expensive, or not clinically feasible. Thus, the consensus also considers the use of other functional methods such as HGS [8]. HGS is a validated tool used to evaluate muscle function in different populations, including CF patients [9,10,11,12,13,14,15]. Legroux-Gérota et al. associated HGS in adults with bone mineral density (BMD) [10]; likewise, Rovedder et al. (in young adults) [11], Bouma, et al. (in adolescents) [13], and Wells et al. (in children) [14] associated it with respiratory function. Notwithstanding the above, studies evaluating muscle strength through HGS and its association with clinical variables with prognostic value are scarce, especially in adults with CF.

Here, our hypothesis is that HGS is a useful tool for nutritional assessment of FFM and that it is associated with parameters of clinical severity.

The objective of our study is to evaluate muscle strength through HGS in clinically stable adults with CF and to determinate if it is associated with the clinical variables of respiratory function, body composition and BMD.

## 2. Material and Methods

A cross-sectional observational study with sequential recruitment of patients who attended the CF outpatient consulting room for their annual examination was conducted between January 2016 and January 2017. The inclusion criteria were: (a) regular follow-up with periodic check-ups at the Cystic Fibrosis Unit of the Regional University Hospital of Malaga and attendance for the annual study at the time of data collection; (b) presentation of diagnostic criteria for CF according to the consensus document published by the Cystic Fibrosis Foundation in 1998; (c) age older than 16 years; (d) having completed pubertal development (Tanner stage V); (e) maintaining a stable clinical condition for at least one month prior to inclusion in the study (no hospital admissions, no respiratory exacerbations requiring intravenous antibiotic therapy and no weight change >3% of body weight); (f) to understand the purpose of the study and sign the informed consent form. All patients who met the inclusion criteria were included in the present study.

### 2.1. Anthropometric and Body Composition Parameters

Weight was assessed through bioimpedance (scale mode, weight function; TANITA MC980MA) and height was obtained by a stadiometer (Holtain limited, Crymuch, UK). With these two values, body mass index (BMI) was calculated.

Dual-energy X-ray absorptiometry (DXA) was performed using a Lunar Prodigy Advance densitometer (General Electric Medical Systems), providing information about total body composition (FFM, fat mass, and BMD) and bone mineral density (BMD) at the level of the lumbar spine (L1–L4) expressed in g/cm^2^; T-score and Z-score were compared with reference values [16]. The diagnosis of low bone mineral density for chronological age or osteopenia/osteoporosis was established according to World Health Organization (WHO) criteria, National Osteoporosis Foundation (NOF) and International Society for Clinical Densitometry (ISCD) guidelines [17,18,19,20]. The software used was EnCore 12.3 (iDXA and Prodigy Advance).

In addition, FFM index (FFMI) was calculated (fat-free mass in kg/height in m^2^) and the prevalence of malnutrition was determined according to European Society for Clinical Nutrition and Metabolism (ESPEN) criteria. Malnutrition according to FFMI was estimated at values < 15 (women) or <17 (men) kg/m^2^ [5].

Muscle strength was assessed using an adult dynamometer (Jamar handgrip dynamometry, Asimow Engineering Co., Los Angeles, CA, USA), and was performed in the non-dominant limb, repeated on three occasions and the mean was recorded. The mean was calculated, and along with the highest value was used to represent HGS. To classify normality, data from the Spanish population were used, establishing the cut-off point at p10 [21]. Data were expressed in absolute figures and compared with the reference population [22].

### 2.2. Dietary Questionnarie

A four-day prospective dietary questionnaire (including one day of the weekend) was fulfilled by all the participants, according to the protocol previously published by our group [23]. The provided data were analysed by means of a computer application designed by our group for this purpose (Dietstat^®^), and the food composition tables of Jiménez and Mataix [24,25] and BEDCA [26] were used. Moreover, the information on vitamin K (VK) was substituted by the USDA database [27].

### 2.3. Assessment of Respiratory Status

The exacerbations recorded during the annual examination were assessed, taking into consideration those happening in the year prior to the evaluation. Such exacerbations were classified into mild/moderate or severe (suggestive symptoms that worsen and require hospitalization and/or intravenous antibiotics on an outpatient basis) [28]. Moreover, patients underwent forced spirometry via a JAEGER pneumotachograph (Jaeger Oxycon Pro® Erich Jaeger, Würzberg, Germany), following the Sociedad Española de Neumología y Cirugía Torácica (SEPAR) guidelines and determining the values of forced vital capacity (FVC), forced expiratory volume in 1 s (FEV1), and the ratio between both (FEV1/FVC). The values were expressed in absolute terms in ml and as percentages according to a reference population [29]. Bronchorrhea, the amount of sputum produced per day expressed in milliliters, was evaluated according to the estimate of the patient during the three days prior to the visit [28].

### 2.4. Statistical Analysis

The Kolmogorov–Smirnov test was used to examine the normality of the quantitative variables, expressed as the mean ± standard deviation. The differences between groups were analyzed by variance analysis adjusted for age. Non-parametric tests (Mann–Whitney) were used when the variables did not follow a normal distribution. The qualitative variables were expressed as proportions, and differences between groups were analyzed via the Chi-square test, using Fisher’s exact test when necessary. The associations of the variables were evaluated by estimating the Pearson or Spearman correlation coefficient, according to normality. For all calculations, a *p* probability of less than 0.05 significant for two tails was considered significant. The data analysis was performed with the SPSS 22.0 program (SPSS Inc., Chicago, IL, USA, 2013).

### 2.5. Ethics

All subjects gave their informed consent for inclusion before they participated in the study. The study was conducted in accordance with the Declaration of Helsinki, and the protocol was approved by the Research Ethics Committee of Malaga Province.

## 3. Results

A total of 53 CF patients were included in the study; 22 (41.5%) were male and 31 (58.5%) were female. Class I and II mutations for the CFTR gene were the most frequently found; for gene A, class I mutation was found in 15.4% [8], class II in 78.8% [30], class III in 1.9% [1], and class IV in 3.8% [2], while no class V mutations were found. For the B gene, the distribution of mutations was class I in 41.7% [20], class II in 39.6% [19], class III in 2.1% [1], class IV in 10.4% [5] and class V in 6.3% [3]. The most frequently found mutation was F508del (67.9%), followed by G542X (7.5%), N1303K (7.5%) and R334W (7.5%), with the remaining mutations being more infrequent. At the time of the study, none of the participants were on treatment with CFTR modulators. Their mean age was 28.3 ± 8.1 years (range 16–67). Exocrine pancreatic insufficiency was present in 75.5% [31] of the patients. Of all the subjects, 49.1% presented FFM malnutrition, accounting for 27.8% of male and 68.4% of female patients. The prevalence of low muscle strength (<p10) was 20.8% [11]. The general characteristics of the patients are displayed in Table 1.

Separating by groups (dynamometry ≥ p10 or <p10), we found that the patients showing lower muscle strength also presented worse respiratory (more total and mild exacerbations, lower FEV1 and FVC), nutritional (lower BMI), and bone status (worse BMD, T-score, and Z-score), as well as a higher percentage of osteoporosis and osteopenia. We also found a non-significant trend towards more bronchorrhea and chronic S aureus and P aeruginosa colonisation (Table 1). However, we found no significant differences between the two groups on the dietary questionnaire.

Patients with FFM malnutrition measured through DXA (FFMI < 17 kg/m^2^ in males and <15 kg/m^2^ in females) presented significantly lower values in HGS compared with normally nourished patients in both mean (35.7 ± 12.1 vs. 25.1 ± 6.8 kg, *p* < 0.01) and maximum (37.2 ± 12.3 vs. 26.6 ± 6.8 kg, *p* < 0.01) dynamometry.

Moreover, a positive correlation was found between mean and maximum dynamometry and age, FVC %, BMI, FFMI (kg/m^2^), FFM (kg), and BMD (Table 2 and Table 3). We found no significance for the dietary questionnaire variables.

## 4. Discussion

In our study, we have found that HGS is associated with respiratory, body composition (FFM), and BMD parameters, making it a useful tool for nutritional and functional assessment in CF adult patients.

Peripheral muscle dysfunction is an important systemic consequence of CF with major clinical implications including malnutrition, fatigability, reduced physical activity, and reduced quality of life. Furthermore, lack of physical activity, intrinsic CF-related factors, inflammation, and metabolic abnormalities increase with disease severity [32,33].

Muscular strength and function can be measured in CF patients through diverse techniques such as assessing the function of the ventilatory muscles via maximal inspiratory pressure (MIP) and maximal expiratory pressure (MEP), 6- or 12-min walk test, squats, vertical jumping ability, HGS, abdominal strength, arm/shoulder strength, quadriceps muscle strength, and stimulating specific muscles (e.g., the thumb abductor muscle) [15,34]. However, due to its simplicity, easy standardization, and low cost, handgrip dynamometry (HGS) has been proposed as a valid tool for nutritional assessment [5,8,35] as it can perform as a surrogate for muscle mass and an indicator of nutritional and functional status. HGS is associated with prognosis in different clinical populations with increased morbidity and mortality [6,7]. In addition, HGS can be more sensitive to changes in nutritional status than the evaluation of FFM through body composition techniques [36,37,38].

The prevalence of low muscle strength (<10th percentile) in our group using HGS was 20.8%. Bouma et al. found an even higher prevalence (40% of individuals aged 6–19 years) using the same criteria [13].

Rovedder et al. found that HGS was associated with FVC and FEV1 in young adults with CF [11]. In the same way, Bouma et al. reported that in a wide sample population of children, adolescents, and young adults HGS was also positively associated with FEV1% [13]. Thus, the individuals who were strong for their age (high HGS) were more likely to have a higher FEV1% than those who were weak for their age; this happened especially in patients with a lower BMI (<50th percentile) [13]. Similarly, in children and adolescents with CF, Sheikh et al. found that FFMI correlated more strongly than BMI to FEV1% [39]. Wells et al. reported that HGS in children was significantly correlated with respiratory function and peak aerobic capacity (by maximal incremental cycling test) while measures of lower body strength were not; they concluded that simple fitness tests such as HGS may be useful indicators of lung health and fitness [14]. Gibson et al. measured HGS in 23 children with CF during a hospital admission for pulmonary exacerbation, then again six weeks posthospitalization, finding that HGS Z-scores increased significantly after hospitalization. Nevertheless, there was no association between HGS Z-scores and FEV1%; a small sample size may have affected the results [38]. In our group, patients with lower muscle strength (HGS < 10th percentile) had a worse respiratory status, presenting more annual exacerbations and lower values of FEV1% and FVC%. Moreover, we found significant positive correlations between HGS and FVC% and a non-significant trend towards more bronchorrhea and chronic S aureus and P aeruginosa colonization [40]. Other authors also reported significant positive associations between other muscle strength measures (inspiratory, quads, shoulders) and respiratory function in children and adults with CF [9,41].

In our study, patients with FFM malnutrition had significantly lower HGS values compared with normally nourished subjects. In addition, we found significant positive correlations between mean and maximum dynamometry and BMI, FFMI, and FFM (kg). Other authors also report significant positive correlations between other muscle strength measurements and FFM in children and adults with CF [9,13]. Patients with CF who are weak for their size may have less FFM, regardless of their BMI, which could negatively impact their respiratory function [13]. Alvarez et al. also reported that an excess of adiposity in adults with CF, particularly in the form of normal-weight obesity associated with low FFM, was inversely associated with respiratory function. Therefore, it is crucial to promote physical activity and an adequate diet in order to improve muscle mass, and also important to monitor the amount of muscle and its function. As HGS is associated with both FFM and respiratory function, this tool behaves as a simple but adequate indicator for nutritional assessment and patient follow-up. Moreover, being an objective measure, it could help motivate patients to engage in physical activity, which increases BMI by building muscle (and not fat mass), reducing sedentary habits, and favouring a healthy diet tailored to their energy and protein requirements [13]. However, in our work we found no association between GSH and protein and fat-soluble vitamin intake.

FFM and muscle strength, along with BMD are independent factors for the prediction of osteoporosis [31,36]. We observed that patients who had an HGS greater than p10 had significantly higher BMD, T-score, and Z-score. We found a higher percentage of patients with osteopenia and osteoporosis in the lower strength group. We also observed significant positive correlations between mean and maximum dynamometry and BMD. Legroux-Gérota et al. also reported that, in a sample population of 55 adults with CF, at the femoral, neck, and total hip BMD values correlated with HGS and the 6-min walk test result. In their group, BMD was low in 78.2% of adults with CF, a figure higher than ours, which was associated with respiratory function, physical function (walking test and HGS), and nutritional status [10]. In addition, Gur et al. observed correlations between BMD and HGS (both at hip and spine level), and FFM, in addition to a high percentage of osteoporosis (27.5%) and osteopenia (37.5%) in 40 CF patients with a mean age of 18 years [30]. In patients with non-CF bronchiectasis, we also found that respiratory parameters, body composition, muscle strength, and bone remodelling biomarkers were associated with a lower BMD [42]. Moreover, in an interventional study on patients with non-CF bronchiectasis, the addition of a hyperproteic oral nutritional supplement enriched with β-Hydroxy-β-Methylbutyrate for pulmonary rehabilitation improved body composition, BMD, and HGS [43]. These results, in a model of chronic respiratory disease similar to CF, evidence the utility of HGS as a surrogate for body composition associated with parameters relevant for patient follow-up.

As strengths of our study, we highlight the use of the HGS normality values of the Spanish population, as well as the assessment of body composition and bone status through DXA (the gold standard in clinical practice), and of respiratory status through different parameters of prognosis in CF (spirometry, bronchorrhea, colonisation). Nonetheless, there are some limitations: it is a cross-sectional study, which impedes drawing conclusions regarding causality; thus, we can only speculate on different associations. Moreover, it is a single-centre study on a moderate number of participants, which poses a greater challenge to generalising of the results.

## 5. Conclusions

In conclusion, the prevalence of low muscle strength in our study was 20.8% as measured by HGS, and as this technique was associated with clinically important parameters for CF patient follow-up (respiratory, body composition (FFM), and BMD), we propose handgrip dynamometry as a tool to be implemented for nutritional assessment in patients with CF. It is simple, requires a short period of time, has a low cost, and is easy to standardize. New prospective studies are still needed in order to evaluate its association with prognosis in terms of morbidity and mortality.

## Figures and Tables

**Table 1:** General characteristics and respiratory, nutritional, and bone status.

		CF	CF		
		Total (*n* = 53)	Dynamometry ≥ p10 (*n* = 42)	Dynamometry < p10 (*n* = 11)	*p*
Age	(m ± SD)	28.3 ± 8.1	30.4 ± 7.6	20.5 ± 4.6	**<0.01**
Sex Male Female	*n* (%)	22 (41.5) 31 (58.5)	17 (40.5) 25 (59.5)	5 (45.5) 6 (54.5)	NS
**RESPIRATORY STATUS**					
Bronchorrhea (mL)	(m ± SD)	20.5 ± 20.2	18.6 ± 20.4	26.5 ± 19.2	NS
Total exacerbations	(m ± SD)	2.3 ± 1.8	2.0 ± 1.5	3.6 ± 2.2	**<0.01**
Severe exacerbations	(m ± SD)	0.41 ± 0.80	0.4 ± 0.8	0.5 ± 0.8	NS
FEV1 (%)	(m ± SD)	63.3 ± 25.6	67.5 ± 25.2	56.6 ± 21.4	**<0.001**
FVC (%)	(m ± SD)	75.3 ± 22.0	79.6 ± 18.2	73.9 ± 23.0	**<0.001**
FEV1/FVC (%)	(m ± SD)	66.7 ± 12.0	68.0 ± 15.7	61.4 ± 17.8	NS
Colonisations					
S. Aureus colonisation	*n* (%)	42 (79.2)	32 (76.2)	10 (90.9)	NS
H. influenzae colonisation	*n* (%)	23 (43.4)	20 (47.6)	3 (27.3)	NS
P. aeruginosa colonisation	*n* (%)	41 (77.4)	31 (73.8)	10 (90.6)	NS
**DIETARY QUESTIONNAIRE**					
Mean calorie intake	(m ± SD)	3444.0 ± 750.6	3324.4 ± 638.0	3683.2 ± 1000.6	NS
Calories/kg of body weight	(m ± SD)	47.5 ± 11.6	45.6 ± 10.6	54.0 ± 5.0	NS
Proteins (%)	(m ± SD)	16.2 ± 2.4	16.1 ± 2.5	16.3 ± 2.6	NS
Proteins/kg of body weight	(m ± SD)	1.9 ± 0.6	1.9 ± 0.6	2.2 ± 0.6	NS
Vitamin A (UI)	(m ± SD)	6202.2 ± 4445.9	5730.0 ± 4616.9	7820.9 ± 3629.5	NS
Vitamin D (UI)	(m ± SD)	3400.4 ± 2855.5	2961.1 ± 2515.1	4906.3 ± 3620.1	NS
Vitamin E (mg)	(m ± SD)	269.3 ± 175.0	261.2 ± 187.5	297.0 ± 131.3	NS
Vitamin K (mcg)	(m ± SD)	1078.5 ± 1596.7	1160.8 ± 1755.7	776.7 ± 778.0	NS
**BONE STATUS**					
BMD	(m ± SD)	1.092 ± 0.237	1.169 ± 0.255	0.991 ± 0.097	**<0.05**
T-score	(m ± SD)	−0.430 ± 1.184	−0.323 ± 1.123	−1.900 ± 1.228	**<0.05**
Z-score	(m ± SD)	−0.449± 1.145	−0.172 ± 1.051	−1.530 ± 0.830	**<0.001**
Normal	*n* (%)	36 (67.9)	32 (76.2)	4 (36.4)	**<0.05**
Osteopenia	*n* (%)	11 (20.8)	8 (19.0)	3 (27.2)	
Osteoporosis	*n* (%)	6 (11.3)	2 (4.8)	4 (36.4)	
**NUTRITIONAL STATUS**					
BMI	(m ± SD)	21.7 ± 3.4	22.1 ± 3.7	20.2 ± 1.8	**<0.05**
Fat mass (kg)	(m ± SD)	15.4 ± 8.1	15.9 ± 8.6	13.4 ± 4.6	NS
Fat-free mass (kg)	(m ± SD)	42.9 ± 9.7	43.1 ± 9.2	42.1 ± 12.2	NS
Fat mass (%)	(m ± SD)	25.9 ± 9.8	26.0 ± 9.7	25.4 ± 11.2	NS
Fat-free mass (%)	(m ± SD)	74.1 ± 9.9	74.0 ± 9.8	74.6 ±11.2	NS
FFMI (kg/talla^2^)	(m ± SD)	16.3 ± 2.5	16.2 ± 2.4	15.8 ± 2.9	NS
Malnourished (FFMI < 17/15)	*n* (%)	26 (49.1)	20 (47.6)	6 (54.5)	
Mean dynamometry	(m ± SD)	30.2 ± 11.3	30.4 ± 11.9	19.5 ± 9.5	**<0.001**
Maximum dynamometry	(m ± SD)	31.7 ± 11.6	33.9 ± 10.9	20.8 ± 9.2	**<0.01**

m ± SD: mean ± standard deviation. CF: Cystic Fibrosis. BMD: Bone mineral density BMI: Body mass index. FEV1: Forced expiratory volume in 1 s. FVC: Forced vital capacity. FFMI: Fat-free mass index. FFMI ≥ 17 V/15 M). Fat-free mass index ≥ 17 in men and ≥15 in women. m ± SD: mean ± standard deviation. NS: not significant. *p*: statistical significance for dynamometry between ≥p10 and <p10. Bold letters indicating the group names or the significant data.

**Table 2:** Correlations between muscle strength and respiratory variables.

	Age	Bronchorrhea	Total Exacerbations	Mild Exacerbations	Severe Exacerbations	FEV1 (%)	FVC (%)	FEV1/FVC (%)
Mean dynamometry (r)	0.334	0.051	−0.067	−0.024	−0.110	0.222	0.302	0.042
*p*	<0.01	NS	NS	NS	NS	NS	<0.05	NS
Maximum dynamometry (r)	0.312	0.072	−0.117	−0.074	−0.135	0.232	0.331	0.057
*p*	<0.05	NS	NS	NS	NS	NS	<0.05	NS

FEV1: Forced expiratory volume in 1 s. FVC: Forced vital capacity. NS: not significant. *p*: statistical significance.

**Table 3:** Correlations between muscle strength and nutritional and bone variables.

	BMD	T-Score	Z-Score	BMI	FFMI	Fat Mass (kg)	Fat-Free Mass (kg)
Mean dynamometry (r)	0.291	0.156	0.272	0.400	0.548	−0.035	0.327
*p*	<0.05	NS	NS	<0.01	<0.001	NS	<0.05
Maximum dynamometry (r)	0.289	0.120	0.246	0.388	0.676	−0.039	0.331
*p*	<0.05	NS	NS	<0.01	<0.001	NS	<0.05

BMI: Body mass index. FFMI: Fat-free mass index. NS: not significant. *p*: statistical significance.
